# Mapping sexual dimorphism signal in the human cranium

**DOI:** 10.1038/s41598-023-43007-y

**Published:** 2023-10-06

**Authors:** Antonietta Del Bove, Lumila Menéndez, Giorgio Manzi, Jacopo Moggi-Cecchi, Carlos Lorenzo, Antonio Profico

**Affiliations:** 1 Department of History and History of Arts, University Rovira i Virgli, Avinguda de Catalunya 35, 43002 Tarragona, Spain; 2 Catalan Institute of Human Paleoecology and Social Evolution (IPHES-CERCA), Zona Educacional 4, Campus Sescelades URV (Edifici W3), 43007 Tarragona, Spain; 3 Department of Anthropology of the Americas, University of Bonn, Oxfordstraße 15, 53111 Bonn, Germany; 4 Department of Evolutionary Biology, University of Vienna, Djerassiplatz 1, 1030 Vienna, Austria; 5 Department of Environmental Biology, Sapienza University of Rome, 00185 Rome, Italy; 6 Department of Biology, University of Florence, Via del Proconsolo, 12, 50122 Florence, Italy; 7 Department of Biology, University of Pisa, Via Luca Ghini, 13, 56126 Pisa, Italy

**Keywords:** Archaeology, Biological anthropology, Computational models

## Abstract

The study of sexual dimorphism in human crania has important applications in the fields of human evolution and human osteology. Current, the identification of sex from cranial morphology relies on manual visual inspection of identifiable anatomical features, which can lead to bias due to user’s expertise. We developed a landmark-based approach to automatically map the sexual dimorphism signal on the human cranium. We used a sex-known sample of 228 individuals from different geographical locations to identify which cranial regions are most sexually dimorphic taking into account shape, form and size. Our results, which align with standard protocols, show that glabellar and supraciliary regions, the mastoid process and the nasal region are the most sexually dimorphic traits (with an accuracy of 73%). The accuracy increased to 77% if they were considered together. Surprisingly the occipital external protuberance resulted to be not sexually dimorphic but mainly related to variations in size. Our approach here applied could be expanded to map other variable signals on skeletal morphology.

## Introduction

Modern humans, compared to other primates, exhibit a lower level of sexual dimorphism^1,2^. Sexual dimorphism consists of phenotypic differences between females and males driven by genetic and physiological factors^3^. The study of sexual dimorphism has a long tradition in forensic anthropology and bioarchaeology. For example, in forensic anthropology the application of laboratory protocols to identify sex permits the reconstruction of the identity of missing persons. In bioarchaeology, classifying sex from skeletal remains belonging to archeological populations is fundamental in reconstructing paleodemography, and it allows for the interpretation of gender-related cultural aspects such as division of labor and funerary behavior^4^.

Among skeletal regions, the pelvis is the most dimorphic anatomical structure in our species, showing a percentage of accuracy in detecting sex close to 100%^5–8^. However, the cranium has been the most frequently prioritized skeletal structure in archaeological and paleoanthropological contexts^9,10^. Several studies^11–14^ have developed methods aiming to increase as much as possible the accuracy in determining sex from the analysis of cranial bones.

Physical anthropologists assign the sex based on the analysis of cranial remains, mainly focusing on differences in morphology of a list of anatomical traits^15–17^. From all three references, the hyperfemale cranial morphology is characterized by the presence of: (i) a smoothed nuchal plane and an external occipital protuberance not developed, (ii) a small and narrow mastoidal process, (iii) a sharp and thin supra-orbital margin beside the concave morphology of the posterior border, (iv) a smoothed glabellar region without anterior projection. In contrast, the hypermale cranial morphology shows: (i) nuchal lines and pronounced external occipital protuberance, (ii) a very large mastoidal process, (iii) a rounded and blunted supra-orbital margin with a flat or inferiorly projected posterior border, (iv) a massive glabellar region with a marked anterior projection.

The scoring system used to characterize sex from cranial morphology takes into account variations in the size and shape of the glabella, mastoidal process, occipital crest, and orbital margin. On average, female individuals are smaller in cranial size and overall more gracile compared to males; however, only a few studies have focused on analyzing variations in cranial size at the interpopulation level^12,18^. In addition, the inclusion of size as a variable in sex identification leads to issues related to the overlapping of the signal due to both sexual dimorphism and cranial allometry.

Biological anthropology has been drastically changed thanks to the introduction of new methods of investigation. Since the foundation of virtual anthropology^19^, we are witnessing an increase in new strategies to identify sex by using geometric morphometrics methods^13,14,20–23^, beside the application of machine/deep learning^12,24–26^, 3D modeling techniques^27,28^ and linear measurements^29,30^.

Most of the recent works focusing on cranial differences between sexes agree that sexual dimorphism signal is more marked if the analyses are performed on single populations than on a worldwide sample^25,31–33^. Despite the vast literature on this topic, protocols used day to day to classify individuals by sex are limited to empirical and visual morphological approaches.

Furthermore, skeletal variation is influenced by additional factors such as subsistence strategies^34,35^ and nutritional patterns that may have a significant impact on level of sex hormones^36^. Within this framework, geographical provenance assumes a pivotal role in increasing the diversification and understanding of the impact of sexual dimorphism on skeletal morphology^37,38^. Numerous studies employing geometric morphometrics have explored sexual dimorphism at population level^39–44^.

In this paper, we apply a landmark-based method to map the signal due to sexual dimorphism in the human cranium of a mixed ancestry sample. Our novelty approach, despite traditional methods in assessing sexual dimorphism on the entire cranial morphology or single bone elements, analyzes the accuracy in discriminating sex at a local level. In that case “local” means a small area defined by 10 contiguous semilandmarks discernible from the bone macro area usually analyzed (Fig. 3). The use of geometric morphometrics methods permits to convert 3D models of human crania into a dense cloud of shape variables acquiring with high precision variations in shape and size. This information is used to build a model at the local level to measure the accuracy in discriminating sexes. In detail, the accuracy is measured by linear discriminate analysis treating each small portion of the cranium as an independent unit. One of the great advantages of studying sexual dimorphism at a local scale is the chance to build self-explained 3D maps of accuracy in detecting sex. We used part of the sample as a training set and the remaining part as a test dataset to avoid issues related to overfitting.

Here, we map the signal of sexual dimorphism on the human cranium using a mixed ancestry sample of 228 individuals. In detail, we test the following hypotheses: 1. Landmark-based methods effectively identify cranial sexually dimorphic traits that align with the anatomical characteristics typically outlined in literature utilizing conventional approaches. Hypothesis 1 is tested by applying linear discriminant analysis on shape and form variables from landmark-based methods. 2. Cranial allometry does not interact with sexual dimorphism. Hypothesis 2 is tested by comparing the allometric trajectories of males and females with differences in shape commonly attributed to sexual dimorphism.

## Results

### Cranial morphology in relation to sexual dimorphism and size

**Figure 1:**
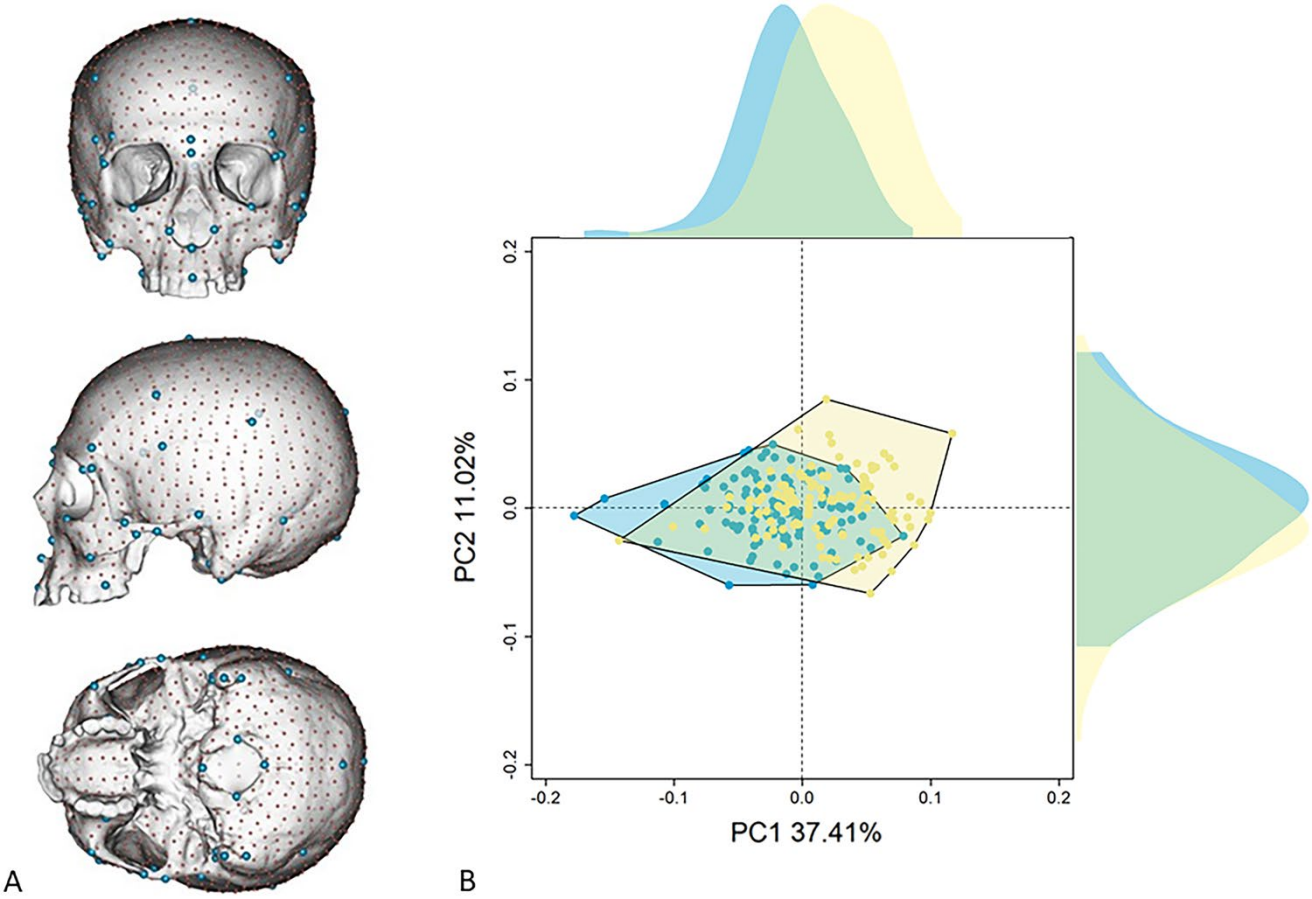
Cranial form in relation to sex. Landmark (in blue) and semilandmark (in red) configurations used to describe cranial variations (**A**); Principal Component Analysis (PCA) of cranial form on female (light blue) and male (yellow) individuals (see the top margin of PCA figures). On the top of the PCA plot, we displayed density plots of PC1 and PC2 pooled by sex (**B**).

We performed a Principal Component Analysis (PCA) on the entire cranial form on sex known individuals belonging to different populations (Table S1). The first two PC scores account for 48.43% of the total variance (PC1 = 37.41%; PC2 = 11.02%) (Fig. 1). The proportion of total variance associated with sex and size is equal to 0.079 and 0.37, respectively. The interaction between sex and size is not statically significant. Along the PC1 female (F) and male (M) individuals are largely overlapped. However, the means of females and males are statistically different (t = −7.12, p-value < 0.001; meanF = −0.020; meanM = 0.019).

**Figure 2:**
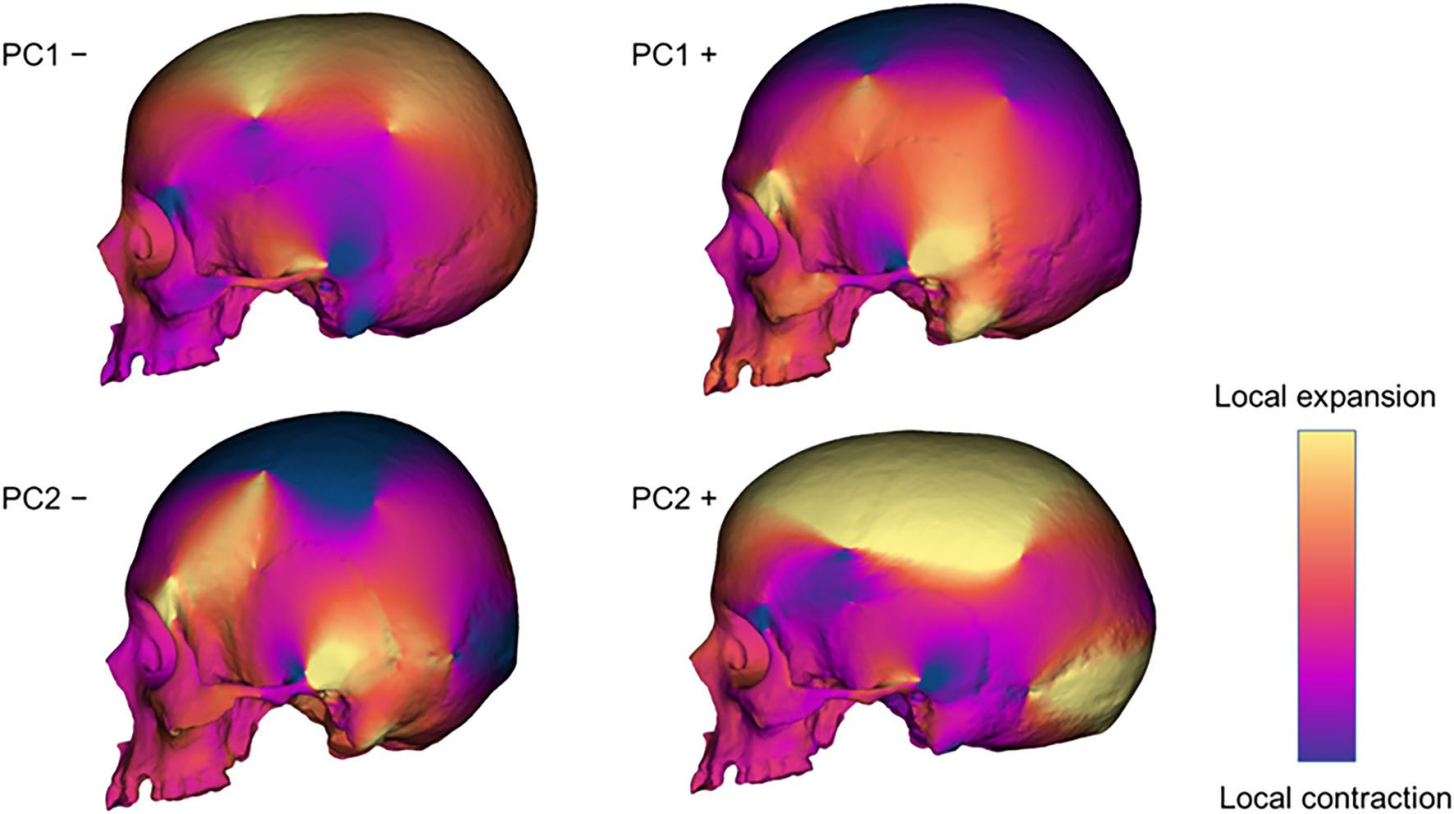
Cranial form variations along PC1 and PC2. Surfaces warping built at the extreme values of the first two principal components. The colors of the form variations represent relative differences in area compared to the mean shape. Warm and cool colors indicate regions that are expanded and contracted, respectively, compared to the mean shape.

The surface warpings built along PC1 show differences mainly related to the pattern of cranial robusticity/gracilization (Fig. 2). At negative values the frontal squama is more horizontally oriented, the morphology of the supraorbital region is less robust, the supero-lateral margin of the orbit is thinner, the zygomatic bone is more gracile in shape, the mastoid processes are smaller and the parietal bones are more rounded. At positive values, we observe an opposite pattern (Fig. 2). Morphological variations detected by PC1 are almost completely related to cranial size (AdjR^2^ = 0.98) and only partially to sexual dimorphism (AdjR^2^ = 0.18).

The PC2 detects differences in the overall cranial architecture (dolichocephaly vs brachycephaly). At negative values, the cranium is shorter and higher; at positive values, the neurocranium is elongated anteroposteriorly (Fig. 2). On PC2 the form variations are not related to sexual dimorphism nor to centroid size.

### Three-dimensional mapping of sexual dimorphism

**Figure 3:**
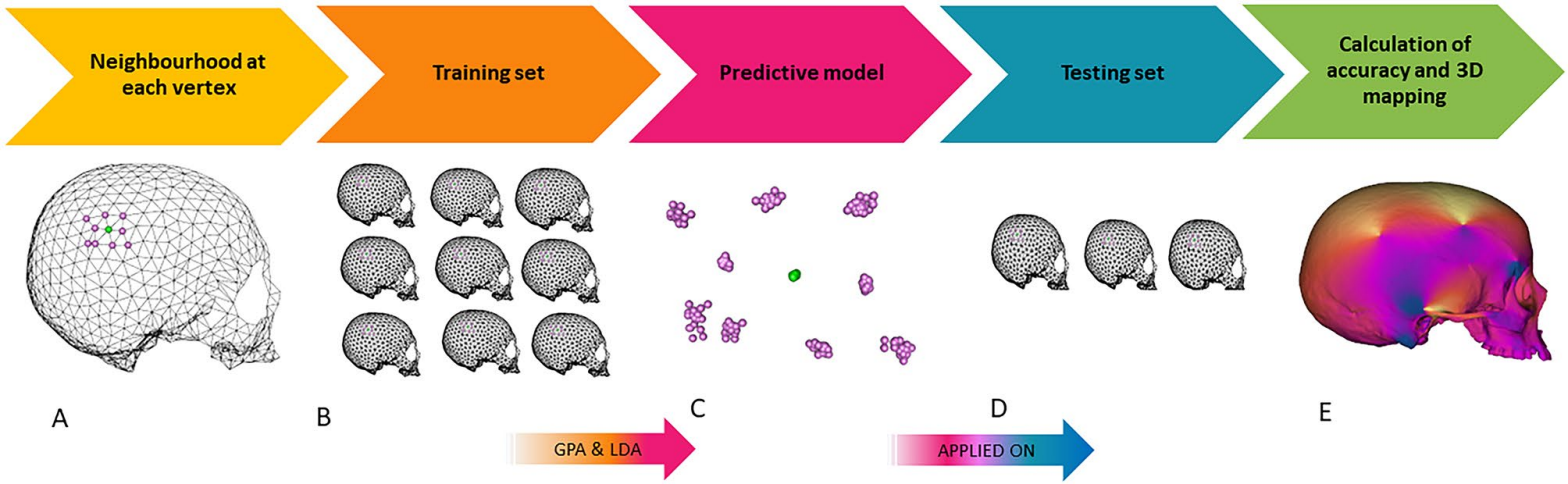
Workflow to map sexual dimorphism: (**A**) calculate a neighborhood of 10 points around each semilandmark. (**B**) Use part of the sample (70%) as a training set. (**C**) Perform generalized procrustes analysis (GPA) on the training set. (**D**) Apply the predictive model to the testing set. (**E**) Calculate accuracy for each set (**B–D**) and repeat the process 100 times, randomly defining the training and testing sets. (**B**-**D**) At the end of the iterative process, calculate the average accuracy value associated with each semilandmark. (**E**) Convert the average accuracy values into a 3D color map.

**Figure 4:**
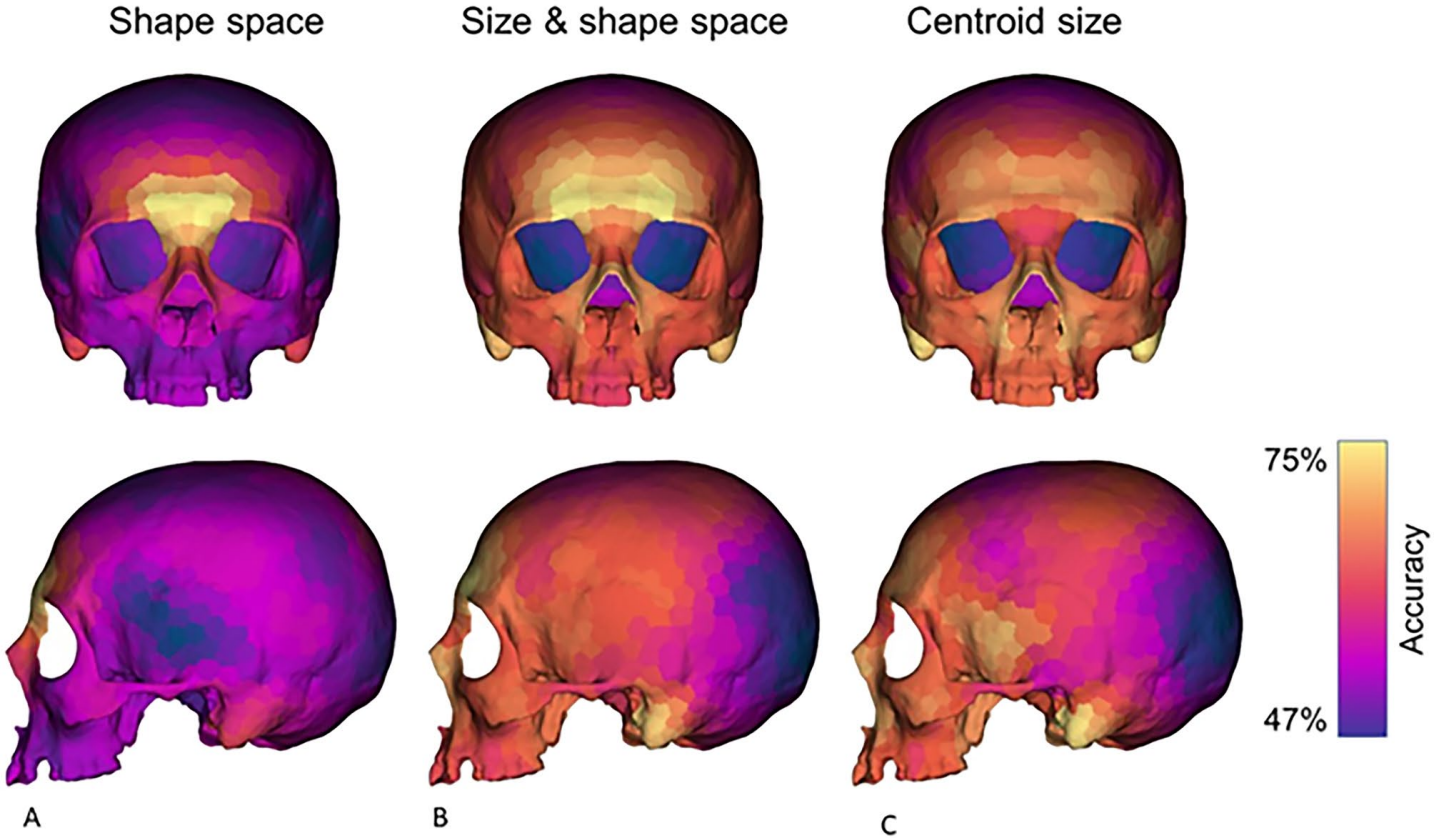
Percentages of accuracy mapped on a 3D model of the cranium. The predictive model has been built using PC scores calculated in the shape (**A**) and the shape-and-size (**B**) space as variables. A third model has been run using only the centroid size (**C**). Warm and cool colors indicate regions that respectively show high and low accuracy in discriminating sex.

3D maps of accuracy were performed by applying Linear Discriminant Analysis (LDA) to define sex as an independent variable, and the PC scores in the shape space and the form space as dependent variables (Fig. 3 and Table 1). A third mapping was performed using the centroid size only as a dependent variable. In the shape space, the central portion of the supraorbital torus, including the glabellar region and part of the frontal squama, resulted to be the most suitable region of the cranium for sexual classification (maximum accuracy equal to 76%) (Fig. 4A). In the shape-and-size space the mastoid processes, the central region of the frontal squama including the supraorbital ridges and the medial portion of the zygomatic bone are the cranial parts showing the highest values of accuracy in classifying sex (accuracy ranged between 71 and 74%) (Fig. 4B). When the centroid size is the only dependent variable used, the mastoid processes and the inferior and lateral portion of the nasal aperture show the highest values of accuracy (ranging between 67 and 70%) (Fig. 4C) (see Table 1 for full results).

**Table 1:** Accuracy values for detecting sex from cranial shape, form and size.

	Shape			Size and shape			Centroid size		
	Tot	F	M	Tot	F	M	Tot	F	M
Entire set	0.66	0.65	0.67	0.73	0.71	0.76	0.71	0.69	0.73
Glabellar and supraciliary region	0.73	0.70	0.77	0.68	0.66	0.72	0.63	0.61	0.66
Frontal bone	0.70	0.68	0.71	0.71	0.68	0.74	0.65	0.63	0.67
Mastoid process	0.64	0.63	0.65	0.73	0.72	0.75	0.70	0.68	0.72
Nasal region	0.67	0.65	0.68	0.73	0.71	0.75	0.63	0.61	0.65
Combined	0.71	0.71	0.71	0.77	0.80	0.75	0.71	0.73	0.70

At each iteration the PC scores are used until the 90% of the variance to define the linear discriminant model (*Tot* entire sample, *F* female, *M* male).

We created a 3D map of accuracy based only on the Italian population to evaluate potential differences based on the structure of the sample under investigation. 3D maps of accuracy based on the Italian population overlap well with the 3D maps built respectively both on the entire sample and the entire sample without the Italian population (Supplementary Fig. S1). According to the previous analysis, in the shape space, the medial-inferior portion of the frontal bone resulted to be the most dimorphic region of the cranium. In the form space, instead, the frontal bone and the mastoid processes are the most dimorphic. Lastly, if only the centroid size is considered, the mastoid processes resulted to be the most dimorphic region of the cranium. These results are confirmed by a correlation test between the average values of accuracy calculated per semilandmark. The correlations between the Italian population and the entire sample in the shape space, form space, and size are all significant and equal respectively to 0.49, 0.85, and 0.84 (Supplementary Fig. S2). In the shape space, when the correlation is calculated by subsetting the values of accuracy by selecting values equal or higher than the third quantile (≥ 0.63), the value of accuracy increases to 0.75. We replicated the same analysis considering as a comparison the entire sample without the Italian population. The correlations between the Italian population and the comparison sample in the shape space, form space, and size are all significant and equal respectively to 0.38, 0.72 and 0.69. In the shape space, the correlation, subsetting the values of accuracy by selecting values equal or higher than the third quantile (≥ 0.63), is equal to 0.74 (Supplementary Fig. S2).

### Geometric morphometric analyses on the most dimorphic cranial regions

**Figure 5:**
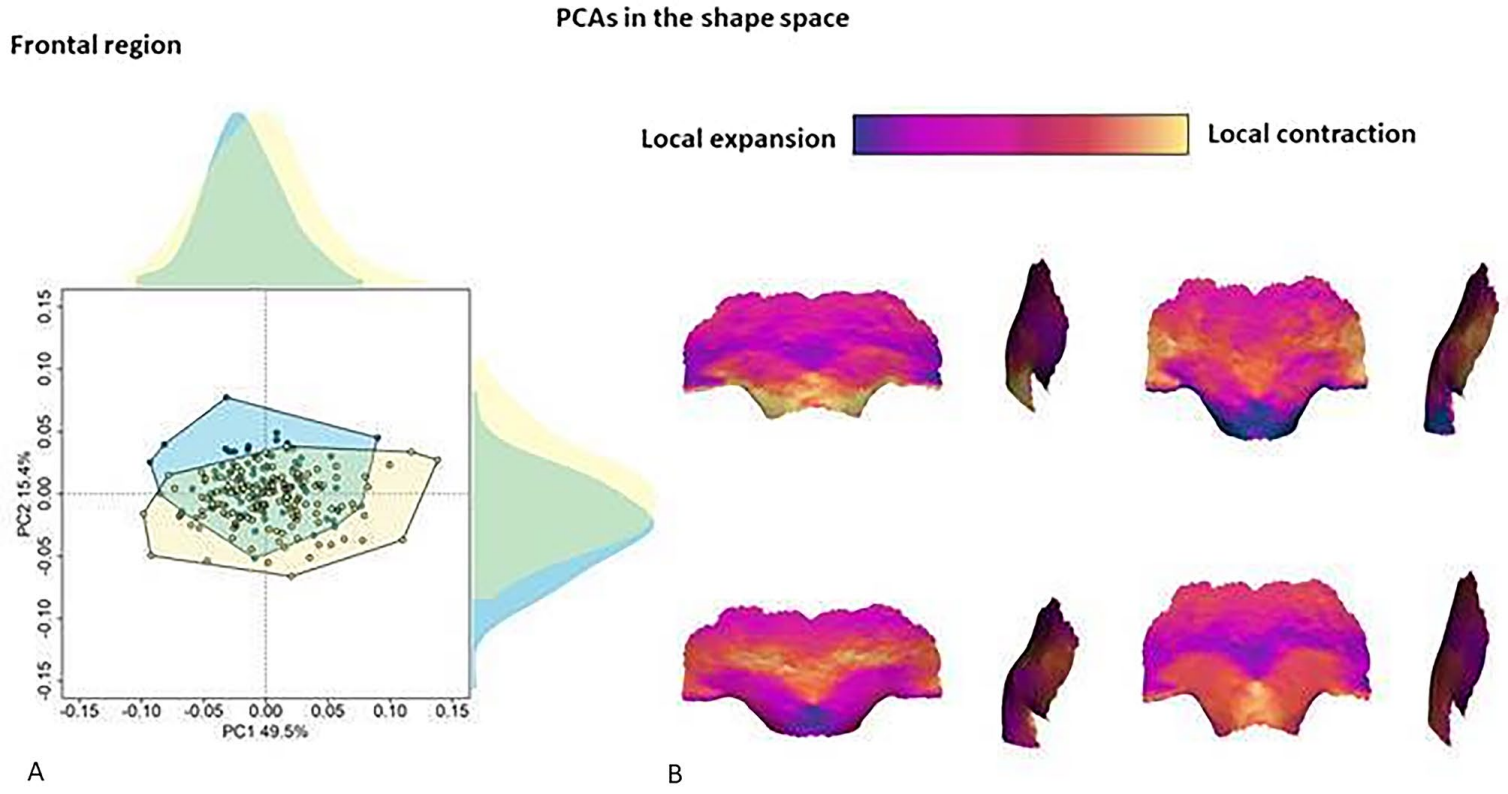
Shape variations of the supraorbital and glabellar regions. Principal component analysis (PCA) of the most prominent dimorphic region identified in the shape space (**A**). In the plot, female and male individuals are represented in light blue and yellow, respectively. The shape variations (**B**) are computed at the extremes of PC1 and PC2. These shape variations are compared to the mean shape: cold and warm colors indicate local contraction and expansion, respectively.

The PCA on the most dimorphic trait identified is glabellar region in the shape space is reported in Fig. 5. The two sexes are slightly separated along PC2 (15.40%), even if the female group is almost completely contained within the male variability (t = 5.32, p-value < 0.001; meanF = 0.007; meanM = −0.007). Variations along PC1 are related to the general structure of the medial-inferior portion of the frontal bone. At negative values, the region of supraorbital torus is narrow and elongated superior-inferiorly. This pattern is opposite at positive values of PC1. Shape variations associated with PC2 are related to sexual dimorphism. At negative values of PC2, the supraorbital ridges are well developed with the presence of supraorbital depression. At positive values, the region in correspondence with the supraorbital torus is flattened, and the upper medial portion of the orbital arch is more rounded.

On average, female individuals possess an expanded glabellar region, and the middle portion of the supraorbital ridges is reduced. On the contrary, in males, the glabellar region is inflated, the central region of supraorbital ridges is more robust and the supraorbital depression well defined.

**Figure 6:**
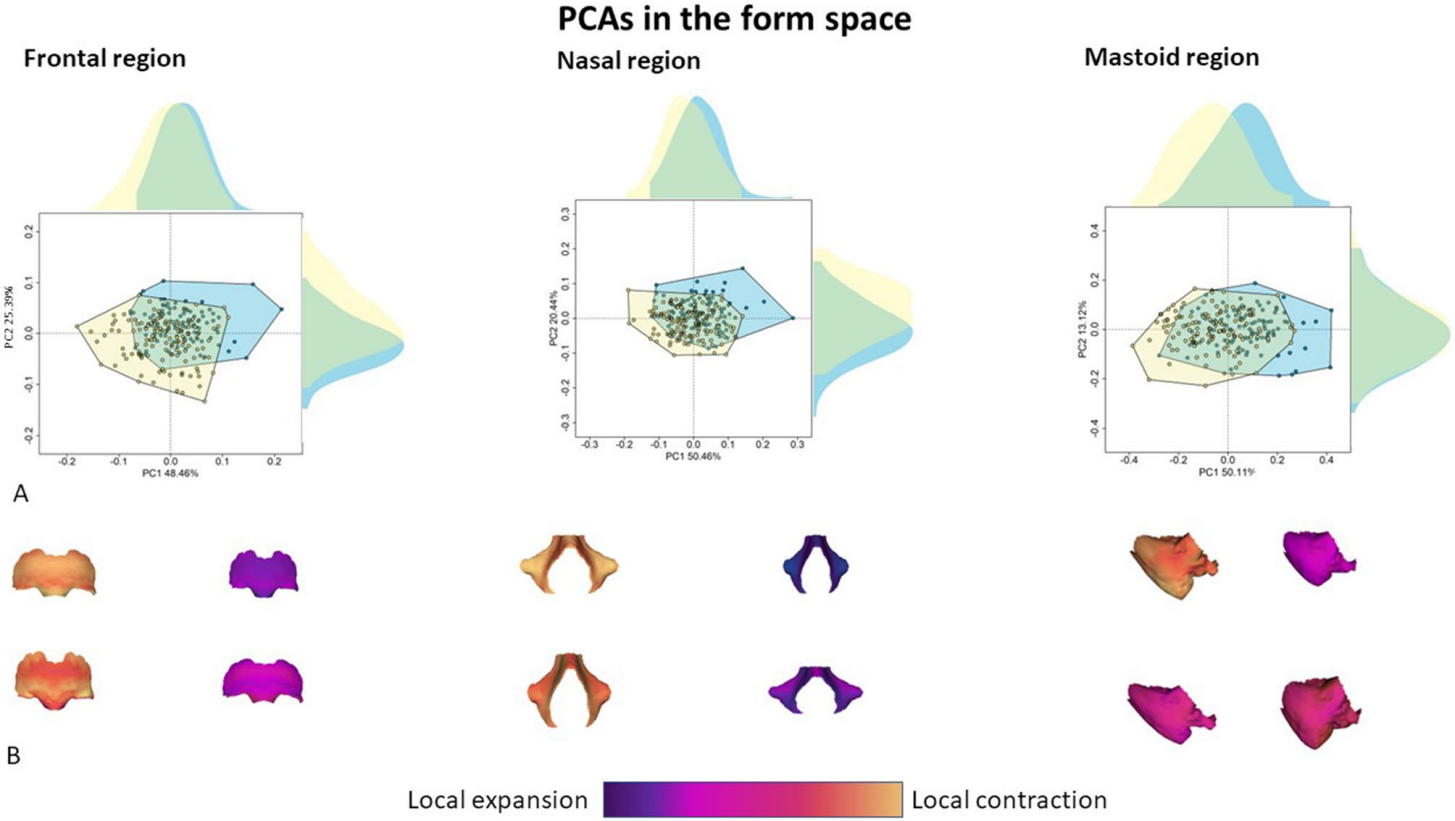
Best dimorphic traits of the human cranium in the form space. Principal component analysis (PCA) of the most prominent dimorphic region identified in the form space (**A**). In the plot, female and male individuals are represented in light blue and yellow, respectively. The form variations (**B**) are computed at the extremes of PC1 and PC2. These form variations are compared to the mean shape: cold and warm colors indicate local contraction and expansion, respectively.

In the form space (Fig. 6), three different cranial modules resulted to be the best in discriminating sex: the inferior portion of the frontal bone, the nasal region, and the mastoid processes. The PCA on the inferior portion of the frontal bone shows variations linked to the general structure of this region. At negative values of PC1 (50.67%), the frontal squama and the upper medial orbital arch are wide and influenced by allometry (R^2^ = 0.93, p-value < 0.001). PC2 (26.41%) detects variations attributable to sexual dimorphism (R^2^ = 0.17, p-value < 0.001). At positive values of PC2: (i) the glabellar region is inflated, (ii) the frontal squama is rounded and (iii) the supraciliary arches are less developed. At negative values of the same principal component, the surface warping shows the presence of the supraorbital region developed with a supratoral sulcus. In addition, at negative values the angle between the medial margin of the orbit and the supraorbital arch is obtuse.

The PCA on the nasal region shows variations mainly linked to the shape and size of the piriform aperture. At negative values of PC1 (50.46%), the piriform aperture is wide, particularly in the middle part. The lateral portion of the nasal region is more enlarged than the medial part. PC2 (20.44%) detects variations associated with the height of the nasal region. At negative values, the nasal aperture is higher and the two lateral margins form a more acute angle. On the contrary, the nasal region is short and wide at positive values. The proportion of variance of PC1 and PC2 associated with sex is equal to 0.06 (p-value z < 0.001) and 0.13 (p-value z < 0.001), respectively.

The PC1 of the PCA performed on the variations in size and shape of the mastoid processes shows a clear pattern linked to allometry (R^2^ = 0.97, p-value z < 0.001). However, about 20% of the variance of PC1 is significantly associated with sex (R^2^ = 0.20, p-value < 0.001) and this proportion of variance is entirely associated also with size. At negative values of PC1, the mastoid process is bigger, and its posterior inferior margin is more expanded. The length of the anterior margin of the mastoid process intercepted by PC2 is not related to sex or to size.

### Sexual dimorphism and cranial allometry in males and females

We analyzed and compared cranial allometric trajectories in males and females by performing multivariate regression on landmark and semilandmark data after Procrustes registration. The centroid size (the square root of the sum of squared distances between each landmark and the centroid) was used as an independent variable and the landmark and semilandmark (after a GPA in the shape and size space) coordinates as dependent variables. The angle between the allometric trajectories in males and females is equal to 10.12°. However, the differences between the trajectories resulted were not statistically significant after performing a permutation test (1000 permutations, p-value = 0.532). Since the angle divergence between the two allometric trajectories is not significant we performed two separate multivariate regressions, one for each biological sex.

**Figure 7:**
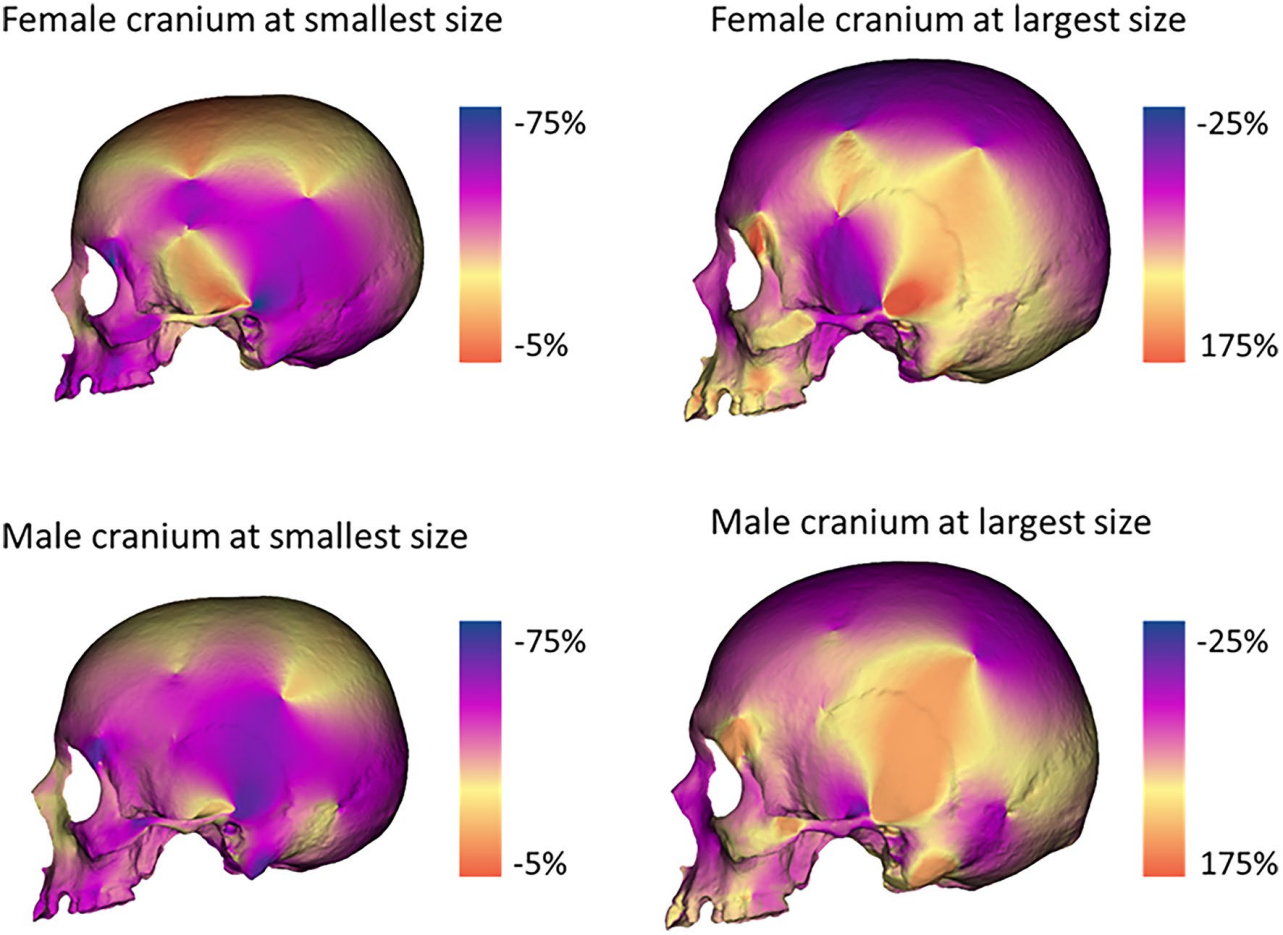
Cranial allometry in females and males. Differences in cranial morphology of the two allometric trajectories. Color maps indicate local variations in area between centroid size extremes in female and male allometric trajectories. Cold colors indicate relative contraction, while warm colors indicate relative expansion.

The allometric trajectories in both sexes shared a similar pattern of form changes from small to large size. From small to large cranial size, the inferior part of the parietal bone (temporal fossa) increases in area as well as the mastoid region (Fig. 7). The expansion of the mastoid process is more accentuated in the male trajectory. At small size the superior part of the cranial calotte is relatively more expanded than at large cranial size. Concerning the facial complex, at small cranial size, the nasal region relatively expanded, as well as the zygomatic process and supraorbital arch. From the posterior view, the region between the lambda and inion is relatively more expanded in males than females at large cranial size^45^.

## Discussion

In this study, we identified the cranial regions presenting a higher signal for sexual dimorphism by performing a series of LDA on small portions of the external surface of the cranium. Each LDA is statistically independent of others since a GPA step is run at each iteration. When the variable cranial size is excluded from the analysis (shape space) the medial-inferior portion of the frontal bone resulted to be the most suitable cranial region for sex classification. Shape variations of this area agree with standard protocols commonly used in sex determination from cranial remains^12,31,46^. In females, the supraorbital torus is less developed, the glabellar region is more expanded, and the angle between the median and superior margin of the orbit is narrower than in males. On the contrary, on average, males possess a robust supraorbital region with a well-defined supratoral sulcus.

The morphological variations of these anatomical features in males and females are the same as described in traditional anthropology protocols^15,46,47^. However, the accuracy of this area is limited to 78.00%. The inclusion of centroid size in LDA analysis reveals which are the three different cranial regions that vary the most in shape and size between the sexes: (i) the medio-inferior portion of the frontal bone, (ii) the nasal cavity and (iii) the mastoid processes. These three regions show respectively 73.00%, 72.00% and 78.00% of accuracy in sex classification.

From the form variation performed on the medial-inferior part of the frontal bone, it is noticeable that in males, the angle between the medial margin of the orbit and the supraorbital arch is acute. This is partially in agreement with standard protocols describing the male orbital shape as less rounded than the one observed in females^22,48^. On average, males exhibit a larger and narrower piriform aperture than females. Nevertheless, this anatomical feature, noted in the literature, is not described in standard protocols as related to sexual dimorphism^13,49^. Concerning the mastoid process, we found, in agreement with standard protocols, that males possess larger mastoid processes^50–54^. However, our results suggest that differences in the mastoid shape are not related to sexual dimorphism.

Our results show that the cranial form is more influenced by size differences than by sexual dimorphism. Allometry dominates the first axis of cranial form variation, and the results of sexual dimorphism change drastically if the variable cranial size is included or excluded from the analysis^55,56^. If the cranial size is included in the analysis, the nasal region and the mastoid processes resulted to be significantly related to sexual dimorphism. The comparison between the allometric trajectories of males and females highlights that they diverge from each other, despite those differences not being statistically significant. More importantly, when the cranial morphology is predicted at a large size in both sexes the external occipital protuberance is well-developed. From standard protocols, this anatomical feature is listed as related to sexual dimorphism probably because on average the male cranium is larger than that observed in females. Therefore, the inclusion of the external occipital protuberance projection in traditional protocols of sex determination may lead to a systematic bias in sex identification, especially when individuals from different populations are being compared. The 3D maps of sexual dimorphism are confirmed by analysing only the individuals from the Italian skeletal collections (see Supplementary Figs. 1 and 2). The vectors of values of accuracy calculated per semilandmark on the Italian population and the entire sample (including and excluding Italian individuals) are statistically significant suggesting no differences in the identification of sexual dimorphic cranial traits in analysing single populations or mixed ancestry samples.

In conclusion, the application of advanced landmark-based methods shows the potentiality of displaying, at a local scale, which regions of the cranium are related to certain factors. Our study confirms that the glabellar region, the supraorbital torus, and mastoid size are sexually dimorphic. However, only the upper and medial portions of the orbital margin differ significantly between sexes. We found that nasal size and nasal shape are different in males and females, and it should be considered when conducting sexual identification analysis. On the contrary, the level of projection of the external occipital protuberance is linked to the cranial size and not strictly to sexual dimorphism.

## Materials and methods

### Sample of study

The total number of individuals analyzed in this study is 228 sex known individuals (112 females and 116 males). The sample consists of different repositories (see Table S1). We considered only adult individuals without any pathology that can affect cranial morphology. In detail, the repositories defining the sample of study are: (i) Lynn Copes Digital Collection from the Anthropology Department—National Museum of Natural History, Washington DC^57^; (ii) Museum of Anthropology “ G. Sergi”—Sapienza University of Rome^58^; (iii) Oloriz Collection—Virtual CT collection of the Museo Nacional de Ciencias Naturales, Universidad Complutense de Madrid; (iv) Anthropological Museum of Florence (University of Florence)^59^; (v) Museum of La Plata (UNLP), Buenos Aires, Argentina^60^ and, the New Mexico Decedent Image Database (NMDID, https://nmdid.unm.edu/)^61^. All the repositories consist of computerized tomography scans with the exception of the photogrammetric models from the repositories of Rome and Florence. The photogrammetric models have been obtained by using the software Agisoft PhotoScan.

### Landmark and semilandmark sets

We acquired ten median landmarks and 20 pairs of bilateral landmarks (Table S2) for a total of 50 landmarks by using 3D Slicer^62^. In addition, we added 500 pairs of bilateral surface semilandmarks to the landmark configuration. We defined the template of surface semilandmarks by applying K-means clustering on the set of vertices of the right side of the cranial surface of a reference specimen (female individual; ID: 4884). Subsequently, the left set of semilandmarks has been specified by applying the function *rotonmat* of the package Morpho^63^, using the cranial landmarks as reference for rigid rotation. After performing the sliding procedure^13^, we symmetrized the entire set of landmarks and semilandmarks to remove the asymmetric component from the analysis. Landmark data has been standardized by applying Generalized Procrustes Analysis (GPA). The average inter-landmark (and semilandmark) distance is equal to 8.73 ± 1.59 mm. To assess the error in the positioning of cranial landmarks, we performed Procrustes ANOVA (according to Fruciano, 2016^64^) by landmarking eight individuals four times each. The repeatability percentage turned out to be equal to 97.87%.

### Multivariate statistics

Geometric morphometric analyses are performed in the shape and form space (the log of centroid size has added a variable to the matrix of shape variables before applying principal component analysis, PCA). Variations among landmark and semilandmark coordinates after GPA have been analyzed by PCA. The accuracy in correctly classifying female and male individuals has been measured by Linear Discriminant Analysis (LDA). We calculated the values of accuracy in each LDA analysis by performing a permutation test, randomly defining each permutation training and testing set. The training and testing sets are represented respectively by 70% and the 30% of the sample. LDA analysis has been performed on (i) the entire set of variables (landmarks plus semilandmarks), (ii) the cranial modules resulted from the automatic mapping of sexual dimorphism, taking into consideration shape, size & shape and size. The last set of analyses includes the application of LDA on the combined modules that emerged from single sessions of the analysis in the shape space, size & shape space and cranial size (see Profico et al., 2019^65^ for methodological specifications). Values of accuracy in detecting sex have been calculated for entire sample and separately for female and male intra-groups. Each value is calculated by running 1000 iterations by defining at each iteration the training (70% of the sample) and testing set (30% of the sample).

Code and R data are available on Zenodo (10.5281/zenodo.8304736)^66^.

### Mapping sexual dimorphism

We propose a landmark-based statistical approach to map a signal associated with a categorical variable (biological sex in this case) onto the landmark configuration. The protocol divides the entire configuration into parts defined by 10 anatomical/geometric points. Each part consists of contiguous points centered on each point of the starting dataset. For each set of 10 points, part of the individuals (70%) is used as a training set and the remaining individuals (30%) as a testing set and the accuracy is evaluated by permutation test. In summary, on each landmark/semilandmark, we define a set of 10 points among landmarks and semilandmarks (1050 sets). For each set of 10 points, the value of accuracy is evaluated by permuting (nperm = 100) individuals in randomly defining training and testing datasets. Each map of sexual dimorphism has requested 105,000 (1050 × 100) LDA analyses.

### Validation of the model at population model

The results from the workflow shown in Fig. 3 has been corroborated by applying the same procedure to the Italian population (sensu lato) here represented by Florentine, Roman, Sardinian anthropological collections. In detail, we calculated the accuracy per semilandmark in classifying sex running 100 iterations on each neighbourhood (total of 1050) splitting the Italian population (130 individuals) into a training (70% of the number of individuals) and testing set (30% of the number of individuals) for a total of 105,000 linear discriminant analysis (100 × 1050). 3D maps of accuracy based on the Italian population have been compared with the 3D maps of the entire sample (including Italian population) and the entire sample without the Italian population. The analysis has been repeated three times in the shape space, size and shape space and by using only the centroid size. Furthermore, we calculated the correlation of the values of accuracy between the Italian population and the entire sample and between the Italian population and the entire sample without the individuals from Italian anthropological collections.

### Supplementary Information

Supplementary Information.

## Data Availability

The authors used different repositories to share different types of data. The Lynn Copes Digital Collection (Ct scan) from the Anthropology Department National Museum of Natural History Washington DC. were deposited at https://www.lynncopes.com/human-ct-scans.html. The NMDID, on the other hand, was deposited in https://nmdid.unm.edu/, which is a database managed by the Office of the Medical Investigator, University of New Mexico that archives and distributes. And, Oloriz Collection—Virtual CT collection of the Museo Nacional de Ciencias Naturales was deposited in Nespos (the project actually expired). As a commitment to transparency and reproducibility, All research data and code publicly available via Zenodo repository (10.5281/zenodo.8304736).
