# Supplementary material for: Mapping sexual dimorphism signal in the human cranium
Source: Sci Rep. 2023 Oct 6;13:16847. doi: 10.1038/s41598-023-43007-y (PMC10558540; doi:10.1038/s41598-023-43007-y)
Supplement: Supplementary file 1 — Supplementary Information. [file 41598_2023_43007_MOESM1_ESM.docx]

**Supplementary information**

**Mapping sexual dimorphism signal in the human cranium.**

Antonietta Del Bove*^1,2^, Lumila Menéndez^3,4^, Giorgio Manzi^5^, Jacopo Moggi Cecchi^6^, Carlos Lorenzo^1,2^, Antonio Profico^7^

Figure S1


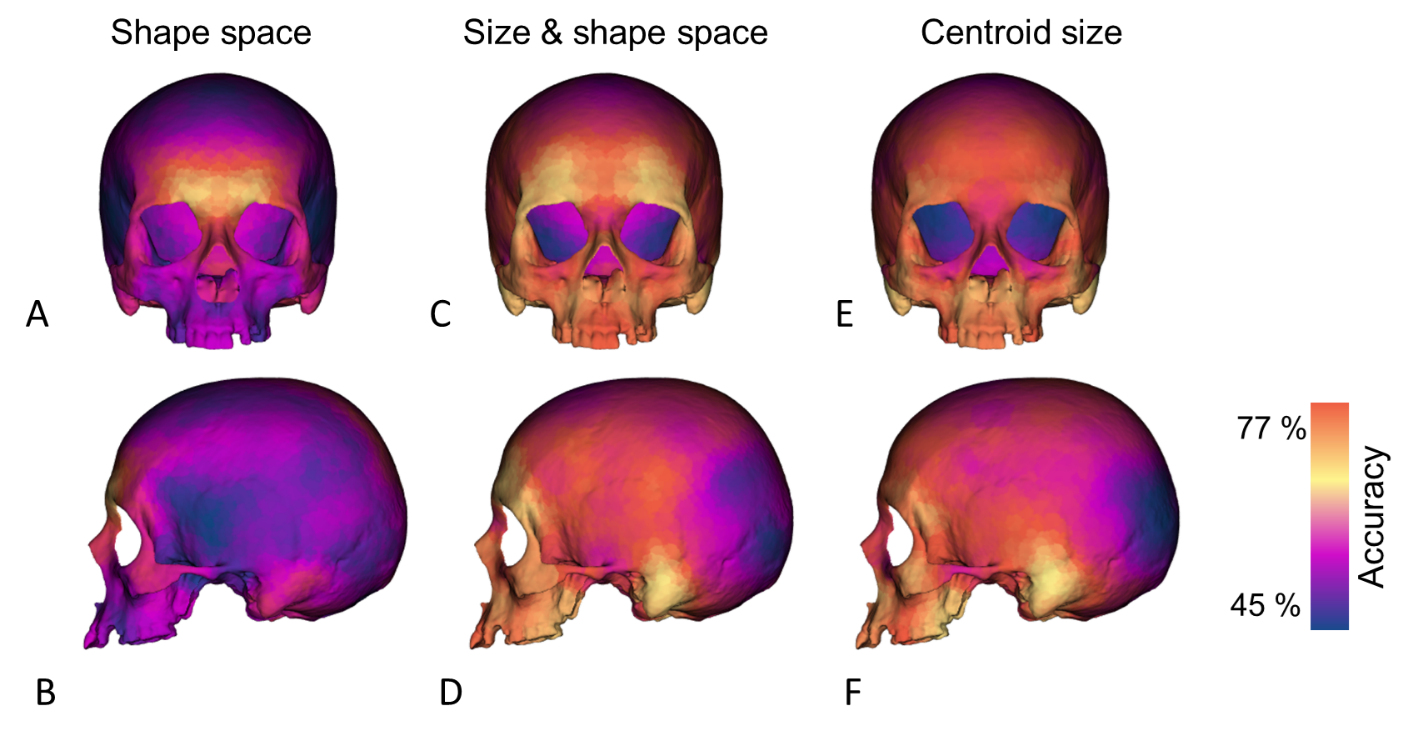


**Fig. S1.** **Percentages of accuracy mapped on a 3D model of the cranium for the Italian sample.** The predictive model has been built using as variables the PC scores calculated in the shape (A, B) and the shape-and-size (C, D) space. A third model has been run using only the centroid size (E, F). Warm and cool colors indicate which regions respectively show high and low accuracy in discriminating sex.

Figure S2


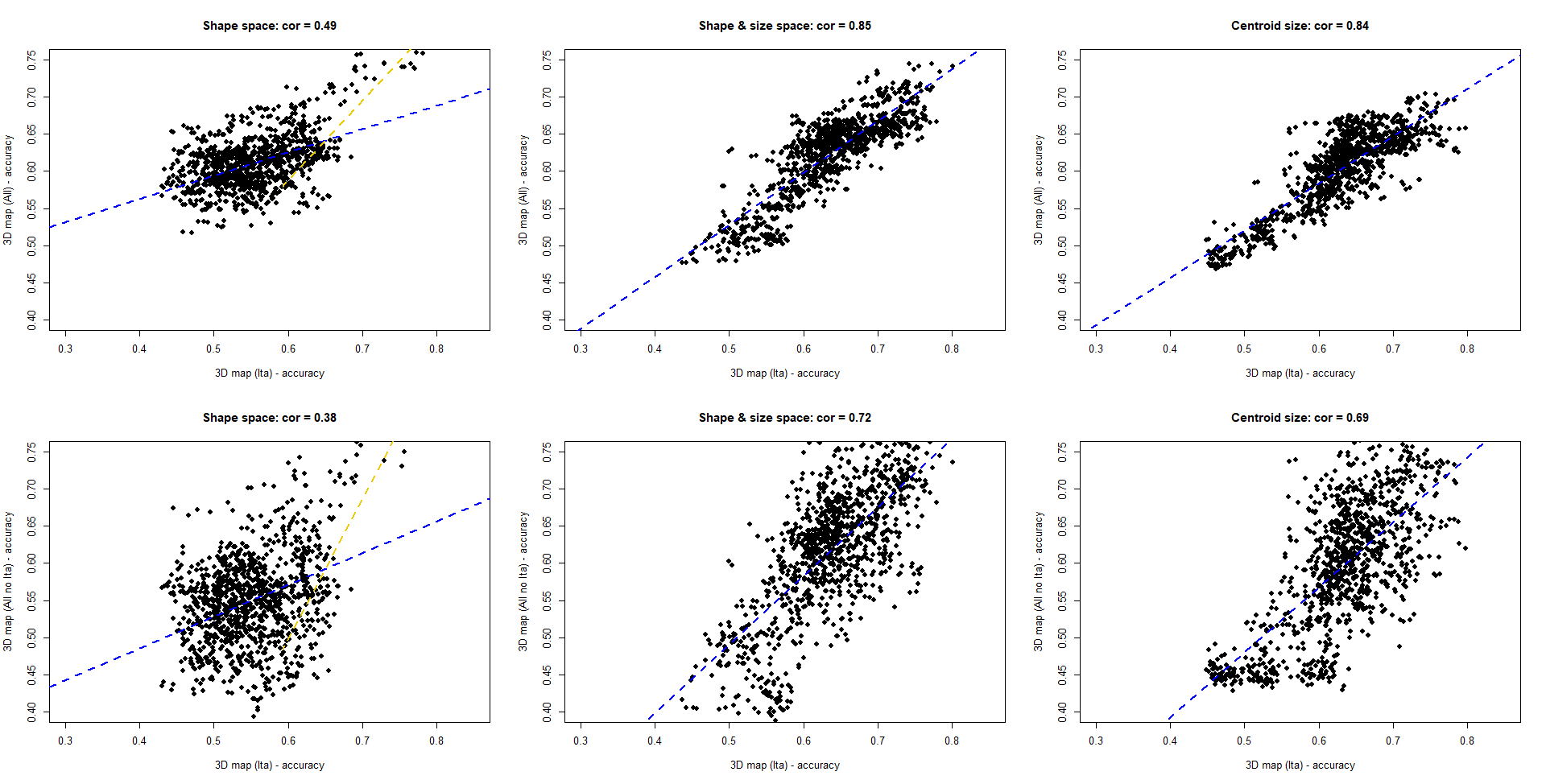


**Fig. S2.** **Correlation analysis between the accuracy values per semilandmark in classifying sex within the Italian population and the full and partial sample.**

First row: correlation plots (shape space on the left, shape, and size space in the middle, centroid size on the right) between the accuracy values within the Italian population (x-axis) and the full sample (y-axis). Second row: correlation plots (shape space on the left, shape and size space in the middle, centroid size on the right) between the accuracy values within the Italian population (x-axis) and the partial sample (excluding Italian individuals) on the y-axis.

Blue and orange dashed lines represent, respectively, the least-squares regression lines calculated on the entire dataset and only on the subset of observations with high accuracy values (75th percentile).

Table S1

Complete study sample

| SEX | ID | Origin | Age of death | | Population | Ancestry |
| --- | --- | --- | --- | --- | --- | --- |
| F | 7 | Italy | | - | Sardinian | European |
| F | 23 | Italy | | - | Sardinian | European |
| F | 131 | Italy | | 24 | Florentine | European |
| F | 165 | Italy | | 23 | Florentine | European |
| F | 348 | South America | | - | Azul | Native American |
| F | 347 | South America | | - | Azul | Native American |
| F | 350 | South America | | - | Azul | Native American |
| F | 351 | South America | | - | Azul | Native American |
| F | 355 | South America | | - | Azul | Native American |
| F | 379 | South America | | - | Azul | Native American |
| F | 1120 | South America | | - | Chubut | Native American |
| F | 1131 | South America | | - | Chubut | Native American |
| F | 1268 | South America | | - | Chubut | Native American |
| F | 17688 | South America | | - | Pampa Grande | Native American |
| F | 17695 | South America | | - | Pampa Grande | Native American |
| F | 17703 | South America | | - | Pampa Grande | Native American |
| F | 17735 | South America | | - | Pampa Grande | Native American |
| F | 405 | Italy | | 30 | Florentine | European |
| F | 414 | Italy | | 30 | Florentine | European |
| F | 418 | Italy | | 26 | Florentine | European |
| F | 730 | Italy | | 34 | Florentine | European |
| F | 840 | North America | | 32 | North America | North America |
| F | 860 | Italy | | 43 | Florentine | European |
| F | 868 | Italy | | 38 | Florentine | European |
| F | 870 | Italy | | 35 | Florentine | European |
| F | 872 | Italy | | - | Florentine | European |
| F | 880 | Italy | | 31 | Florentine | European |
| F | 886 | Spain | | 59 | Spanish | European |
| F | 906 | North America | | 22 | North America | North America |
| F | 921 | North America | | 38 | North America | North America |
| F | 929 | North America | | 20 | North America | North America |
| F | 970 | North America | | 21 | North America | North America |
| F | 100229 | New Mexico | | 31 | no Hispanic | Unknown |
| F | 100263 | New Mexico | | 51 | no Hispanic | Unknown |
| F | 100442 | New Mexico | | 34 | no Hispanic | Unknown |
| F | 100487 | New Mexico | | 28 | no Hispanic | Unknown |
| F | 100516 | New Mexico | | 55 | no Hispanic | Unknown |
| F | 100541 | New Mexico | | 49 | Hispanic Latin | Unknown |
| F | 101106 | New Mexico | | 42 | no Hispanic | Unknown |
| F | 101356 | New Mexico | | 41 | no Hispanic | Unknown |
| F | 101377 | New Mexico | | 24 | no Hispanic | Unknown |
| F | 1015 | North America | | 41 | North America | Unknown |
| F | 101798 | New Mexico | | 50 | no Hispanic | Unknown |
| F | 103668 | New Mexico | | 27 | Native American | Unknown |
| F | 104103 | New Mexico | | 38 | Hispanic Latin | Unknown |
| F | 104125 | New Mexico | | 27 | Hispanic Latin | Unknown |
| F | 104719 | New Mexico | | 42 | Hispanic Latin | Unknown |
| F | 1052 | North America | | 48 | North America | North America |
| F | 105208 | New Mexico | | 31 | Native American | Unknown |
| F | 105294 | New Mexico | | 40 | Hispanic Latin | Unknown |
| F | 105475 | New Mexico | | 42 | Hispanic Latin | Unknown |
| F | 106044 | New Mexico | | 39 | Hispanic Latin | Unknown |
| F | 106212 | New Mexico | | 22 | Hispanic Latin | Unknown |
| F | 1064 | North America | | 33 | North America | Unknown |
| F | 107434 | New Mexico | | 23 | Native American | Unknown |
| F | 108398 | New Mexico | | 37 | Native American | Unknown |
| F | 109148 | New Mexico | | 27 | Native American | Unknown |
| F | 111392 | New Mexico | | 49 | Native American | Unknown |
| F | 1135 | North America | | 62 | North America | North America |
| F | 115842 | New Mexico | | 40 | Native American | Unknown |
| F | 1197 | Spain | | 30 | Spanish | European |
| F | 1199 | Spain | | 39 | Spanish | European |
| F | 1306 | North America | | 36 | North America | North America |
| F | 1333 | North America | | 33 | North America | North America |
| F | 1360 | Italy | | 50 | Florentine | European |
| F | 1370 | Italy | | 20 | Florentine | European |
| F | 1381 | Italy | | 31 | Florentine | European |
| F | 1383 | Italy | | 28 | Florentine | European |
| F | 1384 | Italy | | 30 | Florentine | European |
| F | 1405 | Italy | | 38 | Florentine | European |
| F | 1411 | Italy | | 26 | Florentine | European |
| F | 1419 | Italy | | 26 | Florentine | European |
| F | 1428 | Spain | | 77 | Spanish | European |
| F | 1517 | Italy | | - | Sardinian | European |
| F | 1523 | Italy | | - | Sardinian | European |
| F | 1552 | Italy | | - | Sardinian | European |
| F | 1553 | Italy | | - | Sardinian | European |
| F | 1763 | Italy | | - | Florentine | European |
| F | 1764 | Italy | | 39 | Florentine | European |
| F | 1771 | Italy | | 44 | Florentine | European |
| F | 1777 | Italy | | 24 | Florentine | European |
| F | 1785 | Italy | | 23 | Florentine | European |
| F | 1786 | Italy | | 39 | Florentine | European |
| F | 1790 | Italy | | 20 | Florentine | European |
| F | 2535 | Italy | | - | Roman | European |
| F | 2537 | Italy | | - | Roman | European |
| F | 2538 | Italy | | - | Roman | European |
| F | 2539 | Italy | | - | Roman | European |
| F | 2540 | Italy | | - | Roman | European |
| F | 2541 | Italy | | - | Roman | European |
| F | 2543 | Italy | | - | Roman | European |
| F | 2544 | Italy | | - | Roman | European |
| F | 2545 | Italy | | - | Roman | European |
| F | 2546 | Italy | | - | Roman | European |
| F | 2547 | Italy | | - | Roman | European |
| F | 2548 | Italy | | - | Roman | European |
| F | 2550 | Italy | | - | Roman | European |
| F | 2551 | Italy | | - | Roman | European |
| F | 2552 | Italy | | - | Roman | European |
| F | 2553 | Italy | | - | Roman | European |
| F | 4874 | Italy | | 49 | Syracusan | European |
| F | 4880 | Italy | | 37 | Syracusan | European |
| F | 4883 | Italy | | - | Syracusan | European |
| F | 4884 | Italy | | 32 | Syracusan | European |
| F | 4895 | Italy | | 34 | Syracusan | European |
| F | 5795 | Italy | | - | Sardinian | European |
| F | 5799 | Italy | | - | Sardinian | European |
| F | 5800 | Italy | | - | Sardinian | European |
| F | 5838 | Italy | | - | Sardinian | European |
| F | 5867 | Italy | | - | Sardinian | European |
| F | 5874 | Italy | | - | Sardinian | European |
| F | 5937 | Italy | | - | Sardinian | European |
| M | 1 | Italy | | - | Sardinian | European |
| M | 4 | Italy | | - | Sardinian | European |
| M | 6 | Italy | | - | Sardinian | European |
| M | 15 | Italy | | - | Sardinian | European |
| M | 21 | Italy | | - | Sardinian | European |
| M | 22 | Italy | | - | Sardinian | European |
| M | 258 | Italy | | - | Sardinian | European |
| M | 260 | Italy | | - | Sardinian | European |
| M | 263 | Italy | | - | Sardinian | European |
| M | 265 | Italy | | - | Sardinian | European |
| M | 318 | South America | | - | Azul | Native American |
| M | 319 | South America | | - | Azul | Native American |
| M | 320 | South America | | - | Azul | Native American |
| M | 331 | South America | | - | Azul | Native American |
| M | 1014 | South America | | - | Chubut | Native American |
| M | 1016 | South America | | - | Chubut | Native American |
| M | 1110 | South America | | - | Chubut | Native American |
| M | 1111 | South America | | - | Chubut | Native American |
| M | 1112 | South America | | - | Chubut | Native American |
| M | 1122 | South America | | - | Chubut | Native American |
| M | 17697 | South America | | - | Pampa Grande | Native American |
| M | 17739 | South America | | - | Pampa Grande | Native American |
| M | 412 | Italy | | 20 | Florentine | European |
| M | 419 | Italy | | 33 | Florentine | European |
| M | 734 | Italy | | 25 | Florentine | European |
| M | 735 | Italy | | 35 | Florentine | European |
| M | 794 | Spain | | - | Spanish | European |
| M | 830 | North America | | 28 | North America | North America |
| M | 855 | Italy | | 36 | Florentine | European |
| M | 864 | North America | | 42 | North America | North America |
| M | 869 | Spain | | 42 | Spanish | European |
| M | 874 | Italy | | 22 | Florentine | European |
| M | 878 | Italy | | 36 | Florentine | European |
| M | 100099 | New Mexico | | 50 | Hispanic Latin | Unknown |
| M | 100144 | New Mexico | | 47 | no Hispanic | Unknown |
| M | 100148 | New Mexico | | 58 | Hispanic Latin | Unknown |
| M | 100169 | New Mexico | | 20 | no Hispanic | Unknown |
| M | 100205 | New Mexico | | 43 | no Hispanic | Unknown |
| M | 100221 | New Mexico | | 34 | Hispanic Latin | Unknown |
| M | 100284 | New Mexico | | 47 | Hispanic Latin | Unknown |
| M | 100309 | New Mexico | | 37 | no Hispanic | Unknown |
| M | 100371 | New Mexico | | 46 | Hispanic Latin | Unknown |
| M | 100389 | New Mexico | | 36 | Hispanic Latin | Unknown |
| M | 100418 | New Mexico | | 35 | no Hispanic | Unknown |
| M | 100543 | New Mexico | | 25 | Native American | Unknown |
| M | 100967 | New Mexico | | 33 | no Hispanic | Unknown |
| M | 101121 | New Mexico | | 50 | Native American | Unknown |
| M | 101378 | New Mexico | | 47 | Hispanic Latin | Unknown |
| M | 101599 | New Mexico | | 26 | Hispanic Latin | Unknown |
| M | 102436 | New Mexico | | 37 | Native American | Unknown |
| M | 102965 | New Mexico | | 23 | Native American | Unknown |
| M | 103044 | New Mexico | | 39 | Native American | Unknown |
| M | 103205 | New Mexico | | 29 | Native American | Unknown |
| M | 103634 | New Mexico | | 43 | Native American | Unknown |
| M | 103640 | New Mexico | | 31 | Native American | Unknown |
| M | 1068 | Spain | | 48 | Spanish | European |
| M | 1112 | Spain | | 23 | Spanish | European |
| M | 1168 | North America | | 33 | North America | North America |
| M | 1187 | Spain | | 40 | Spanish | European |
| M | 1192 | Spain | | 67 | Spanish | European |
| M | 1280 | North America | | 75 | North America | North America |
| M | 1282 | Spain | | 66 | Spanish | European |
| M | 1293 | North America | | 51 | North America | North America |
| M | 1363 | Italy | | 44 | Florentine | European |
| M | 1368 | Italy | | 66 | Florentine | European |
| M | 1385 | Italy | | 26 | Florentine | European |
| M | 1386 | Italy | | 28 | Florentine | European |
| M | 1387 | Italy | | 27 | Florentine | European |
| M | 1393 | Italy | | 20 | Florentine | European |
| M | 1395 | Italy | | 24 | Florentine | European |
| M | 1399 | Italy | | 21 | Florentine | European |
| M | 1400 | Italy | | 23 | Florentine | European |
| M | 1403 | Italy | | 30 | Florentine | European |
| M | 1406 | Italy | | 18 | Florentine | European |
| M | 1410 | Italy | | 57 | Florentine | European |
| M | 1412 | Italy | | 35 | Florentine | European |
| M | 1428 | Italy | | 35 | Florentine | European |
| M | 1429 | Italy | | 36 | Florentine | European |
| M | 1430 | Italy | | 60 | Florentine | European |
| M | 1432 | Italy | | 35 | Florentine | European |
| M | 1433 | Italy | | 50 | Florentine | European |
| M | 1505 | Italy | | - | Sardinian | European |
| M | 1507 | Italy | | - | Sardinian | European |
| M | 1508 | Italy | | - | Sardinian | European |
| M | 1518 | Italy | | - | Sardinian | European |
| M | 1525 | Italy | | - | Sardinian | European |
| M | 1533 | Italy | | - | Sardinian | European |
| M | 1535 | Italy | | - | Sardinian | European |
| M | 1537 | Italy | | - | Sardinian | European |
| M | 1756 | Italy | | 41 | Florentine | European |
| M | 1770 | Italy | | 44 | Florentine | European |
| M | 1773 | Italy | | 24 | Florentine | European |
| M | 1774 | Italy | | 43 | Florentine | European |
| M | 1781 | Italy | | 50 | Florentine | European |
| M | 1789 | Italy | | 23 | Florentine | European |
| M | 2524 | Italy | | - | Roman | European |
| M | 2525 | Italy | | - | Roman | European |
| M | 2526 | Italy | | - | Roman | European |
| M | 2527 | Italy | | - | Roman | European |
| M | 2528 | Italy | | - | Roman | European |
| M | 2529 | Italy | | - | Roman | European |
| M | 2530 | Italy | | - | Roman | European |
| M | 2531 | Italy | | - | Roman | European |
| M | 2532 | Italy | | - | Roman | European |
| M | 2533 | Italy | | - | Roman | European |
| M | 4835 | Italy | | 60 | Syracusan | European |
| M | 4839 | Italy | | 64 | Syracusan | European |
| M | 4843 | Italy | | 56 | Syracusan | European |
| M | 4848 | Italy | | 66 | Syracusan | European |
| M | 4849 | Italy | | - | Syracusan | European |
| M | 4850 | Italy | | 64 | Syracusan | European |
| M | 4852 | Italy | | 36 | Syracusan | European |
| M | 4858 | Italy | | 28 | Syracusan | European |
| M | 4861 | Italy | | 19 | Syracusan | European |
| M | 4862 | Italy | | 21 | Syracusan | European |
| M | 4882 | Italy | | 36 | Syracusan | European |

Table S2

Table 2. List of cranial landmarks used in this study.

| Numbers | Landmarks | Definition |
| --- | --- | --- |
| 1 | Prosthion | The most anterior point in the midline on the alveolar process of the maxillae. |
| 2 | Nasospinale | The point where a line drawn between the inferior most point of the nasal aperture crosses the midsagittal plane. |
| 3 | Nasion | The point of intersection between the frontonasal suture and the midsagittal plane. |
| 4 | Glabella | The most anterior midline point on the frontal bone, usually above the frontonasal suture. |
| 5 | Bregma | The ectocranial midline point where the coronal and sagittal sutures intersect. |
| 6 | Lambda | The ectocranial midline point where the sagittal and lambdoid sutures intersect. |
| 7 | Opisthocranion | Instrumentally determined most posterior points of the skull not on the external occipital protuberance. |
| 8 | External occipital protuberance crest | Is a highly variable median line or crest that passes between the right and the left nuchal musculature. |
| 9 | Opisthion | Midline point at the posterior margin of the foramen magnum. |
| 10 | Basion | Midline point on the anterior margin of the foramen magnum. |
| 11-12 | Zygomatic process inf. | Inferior point of zygomatic process. |
| 13-14 | Zygomatic process sup. | Superior point of zygomatic process. |
| 15-16 | Alare | The most lateral points on the nasal aperture in a transverse plane. |
| 17-18 | Ectoconchion | The intersection of the most anterior surface of the lateral border of the orbit. |
| 19-20 | Frontomalare temporale | The most laterally positioned point of the fronto-malar suture. |
| 21-22 | Frontotemporale | The point where the temporal line reaches its most anteromedial position on the frontal. |
| 23-24 | Eurion | Ectocranial points on opposite sides of the skull that form the termini of the line of greatest cranial breadth. |
| 25-26 | Inferior temporal process | Inferior point where the zygomatic process joints with the temporal bone to form the zygomatic arch. |
| 27-28 | Superior temporal process | Superior point where the zygomatic process joints with the temporal bone to form the zygomatic arch. |
| 29-30 | Stephanion | Point where the upper temporal line cuts the coronal suture. |
| 31-32 | Pterion | Is located on the side of the skull, just behind the temple, where the frontal, parietal, temporal and sphenoid bones join together. |
| 33-34 | Foramen magnum lateral point | Most lateral points on the foramen magnum aperture in a transverse plane. |
| 35-36 | Zygion | Most lateral points on the zygomatic arch. |
| 37-38 | Ectomolare | Most lateral points on the outer surface of the alveolar borders of the maxilla. |
| 39-40 | Mastoid notch | Most inferior point on juxtamastoid crests. |
| 41-42 | Auricolare | Point on the lateral aspect of the root of the zygomatic process at the deepest incurvature. |
| 43-44 | Mastoid | Extreme point mastoid process. |
| 45-46 | Petrotympanic crest | Lateral origin of petrotympanic crest. |
| 47-48 | Acoustic meatus | Internal acoustic meatus. |
| 49-50 | Asterion | The posterior point end of the parietomastoid suture. |
